# Interventions to improve equational reasoning: replication and extension of the Cuisenaire-Gattegno curriculum effect

**DOI:** 10.3389/fpsyg.2023.1116555

**Published:** 2023-08-28

**Authors:** Ian Benson, Nigel Marriott, Bruce D. McCandliss

**Affiliations:** ^1^School of Education, University of Roehampton, London, United Kingdom; ^2^Ian Benson and Partners Ltd., London, United Kingdom; ^3^Marriott Statistical Consulting Ltd., Bath, United Kingdom; ^4^Graduate School of Education, Stanford University, Stanford, CA, United States

**Keywords:** aptitude-treatment interactions, arithmetic fluency, pre-algebra, Cuisenaire-Gattegno, Cuisenaire rods

## Abstract

**Introduction:**

The ability to reason about equations in a robust and fluent way requires both instrumental knowledge of symbolic forms, syntax, and operations, as well as relational knowledge of how such formalisms map to meaningful relationships captured within mental models. A recent systematic review of studies contrasting the Cuisenaire-Gattegno (Cui) curriculum approach vs. traditional rote schooling on equational reasoning has demonstrated the positive efficacy of pedagogies that focus on integrating these two forms of knowledge.

**Methods:**

Here we seek to replicate and extend the most efficacious of these studies (Brownell) by implementing the curriculum to a high degree of fidelity, as well as capturing longitudinal changes within learners via a novel tablet-based assessment of accuracy and fluency with equational reasoning. We examined arithmetic fluency as a function of relational reasoning to equate initial performance across diverse groups and to track changes over four growth assessment points.

**Results:**

Results showed that the intervention condition that stressed relational reasoning leads to advances in fluency for addition and subtraction with small numbers. We also showed that this intervention leads to changes in problem solving dispositions toward complex challenges, wherein students in the CUI intervention were more inclined to solve challenging problems relative to those in the control who gave up significantly earlier on multi-step problems. This shift in disposition was associated with higher accuracy on complex equational reasoning problems. A treatment by aptitude interaction emerged for both arithmetic equation reasoning and complex multi-step equational reasoning problems, both of which showed that the intervention had greatest impact for children with lower initial mathematical aptitude. Two years of intervention contrast revealed a large effect (d = 1) for improvements in equational reasoning for the experimental (CUI) group relative to control.

**Discussion:**

The strong replication and extension findings substantiate the importance of embedding these teaching aides within the theory grounded curricula that gave rise to them.

## 1. Introduction

In an earlier paper we described the results of a systematic review of research into the efficacy of Cuisenaire rods and the Cuisenaire-Gattegno (Cui) approach to equational reasoning (Benson et al., 2022). The present replication-extension study investigated four questions motivated by those observations. Firstly, we expected to find medium to large effects of Cui compared to a wide range of traditional approaches. One of the highest-fidelity studies was due to William Brownell, U.C. Berkeley, who designed an unusual longitudinal experiment to investigate the efficacy of Cui. Secondly, we explored Brownell's suggestion that the treatment effect might be tightly coupled with the duration of the intervention and the consistency of its application. Thirdly, Brownell presented evidence for an aptitude-treatment interaction in that the effects would be greater for children with lower scholastic aptitude. And finally, he proposed, but didn't test, an hypothesis that Cui would be an excellent preparation for future learning.

In Section 2.1 we outline a co-design framework for mathematical instruction and assessment developed with teachers and describe how it was adapted to experiment design. Section 2.2 discusses how we extended Brownell's post-test design to perform a replication-extension study with two English primary schools over two years. In seeking to replicate his attribute-treatment interaction we adopt a language neutral test of domain general reasoning in place of Brownell's original verbal reasoning test of scholastic aptitude. Ours is pre-post-test but with non-experimental treatment and control. It is non-experimental in that we had a single school in each arm of the study which was not randomly selected. This is a common feature of the pre-post-tests in the systematic review. We adapt Brownell's approach in our statistical analysis. Finally, to test his future learning hypothesis, we look both at gains recorded during Cui training and gains recorded in the six months following the end of the intervention. Our results are reported in Section 3.

Section 4 discusses the contribution of this work, its limitations and next steps. An online Appendix provides Supplementary material. It reproduces the CUI equational reasoning test item data and describes the core 80-unit lesson sequence we prepared with teachers for their intervention. The earlier paper reproduced a flow chart of the Cui approach to coordinating vision, audition, haptic, sensorimotor and introspective modalities. It is annotated in the Appendix to illustrate how following this curriculum unfolding can lead to the construction of physical and mental models for numbers in some chosen base.

### 1.1. Theoretical background

Our earlier paper discussed the construct of equational reasoning as it is encountered in mathematics and in mathematics education pedagogy (Benson et al., 2022). *Equational reasoning (ER)*, operating on equations, includes substituting equivalent expressions within part of an equation. *Reasoning about equivalence*, a refinement of ER, is a repetitive symbolic procedure where algebraic expressions, or terms, within an expression are replaced by an equivalent until a “solved” form is reached.

There are two approaches to instruction in ER. In an influential paper, Skemp (1976) contrasts these as *relational* vs. *instrumental* understanding. Relational understanding means “knowing what to do and why.” Instrumental understanding—the possession of a rule and the ability to use it—commonly passes as understanding for many pupils and their teachers.

An example of instrumental reasoning might be drill and practice with “number bonds,” or the conventionally rehearsed “place-value” procedure as a way of multiplying two numbers with several digits. A relational approach to this activity, as advanced by Cui, would be to construct a 2 or 3 dimensional physical or mental model of the situation. This is then brought into harmony with the symbolic polynomial expansion of the numbers concerned within a chosen number base.

An over emphasis in pedagogy on instrumental rather than relational understanding often leads to chronic under achievement. In our systematic review of the Cui approach we explored how construction with Cuisenaire rods advanced relational pedagogy. We found that the contrast introduced by using the rods to support relational reasoning produced significant effects in the meta-analysis. However there was substantial variability in results which we wanted to better understand. What we learnt was that the physical manipulative component of the original approach—the rods themselves—have had a greater adoption than efforts to retain or adapt its curriculum elements. Curriculum design fidelity captured a significant proportion of the variability in efficacy reported in the meta-analysis.

In designing the present study we set out to replicate and extend the experiment with the strongest results conducted by Brownell (1967b). To explain our findings we offer a novel category theoretic model for the Cui approach to equational reasoning. In recent years category theory, or conceptual mathematics has been developed as a more or less ubiquitous component of pure mathematics, with applications from theoretical physics to computer science (Lawvere and Schanuel, 2004; Cheng, 2022).

#### 1.1.1. A category theoretic model of the Cui approach to equational reasoning

In the Cui approach students directly manipulate objects such as rods and named drawings, whose elements obey structural rules and whose names as objects obey algebraic grammar rules (Gattegno, 1963a; Benson, 2011; Goutard, 2017; Abreu-Mendoza et al., 2021). “All the operations with integers and fractions can be studied simultaneously (with colored rods); whole numbers being recognized as the equivalence class of their partitions and fractions as ordered pairs, one serving to measure the other, or as operators (functions) belonging to classes of equivalence which are the rational numbers involved in the operations” (Fedon, 1966, p. 201). Learners experience number in the form of the category of finite sets whose structure is expressed in their 3D constructions, 2D diagrams and in equational reasoning. As Davydov (1975) notes “‘Set' here is a *strictly theoretical term* ... At present the point of departure for this system (in the mathematics curriculum) is ‘relation or structure.' The problem of finding a means of presenting and explaining this system to children seven or eight years old is really the problem of finding the ‘beginning' of the mathematics course.”

Gattegno's search for the “beginning of mathematics” led him to observe “each of us (learnt) to talk before or around the age of two. The mental equipment for mastering this skill must actually exist and it proves its existence by functioning... One of the difficulties (in modeling this mathematically) resides in the fact that the grasp of meanings precedes verbalisation and that words per se are not the message, but only one of the possible vehicles for the message.. To have a working model—a mathematics—it is necessary to ... reach the way in which meanings select their own expressions and place them adequately in the flow of speech in order to provide, through a set of *transformations*, the required equivalences. It is my hunch today that *equivalence*, which carries within itself the dynamic component of transformation, is the cardinal concept of mathematics. Perhaps one day I shall be able to tell the story of ‘equivalence through transformation' and its place in the study of speech” (Gattegno, 1970, p. 136). Dehaene, like Gattegno, argues that from birth, the child's brain must already possess two key ingredients: “all of the machinery that makes it possible to generate a plethora of abstract formulas (a combinatorial language of thought) and the ability to choose from these formulas wisely, according to their plausibility given the data” (Dehaene, 2020, p. 43) . Dehaene et al. (2022) note that “whether they possess recursive programs has not yet been tested.”

In Cui arithmetic sum is modeled as a 2D “diagram” of end to end rods—a so-called “train.” It models coproduct as a disjoint set union. Rods stacked as 3D “crosses” and “towers” model products. Associativity follows from the observation that these constructions are independent of the sequence in which any two ends of rods (sides of rods) are juxtaposed into their adjacent places (spaces). Commutativity of addition follows from the observation that the length of a train depends only on the combination of rods, rather than their permutation. Commutativity of product follows by noticing that a train of *ulotsofv* rods [both measured with the same unit rod, *w* (say)] is equivalent in length to one composed of *vlotsofu* rods (measured in the same way). It is more common in the literature to illustrate commutativity of ‘*ulotsofv*' by constructing two rectangles: (*u, v*) (say) with *u* measured with *w* copies of *v* side by side and (*v, u*). We can then demonstrate that the two rectangles are congruent. However “equivalent area” does not generalize in the way we require to commutativity of a tower of more than two rods. We need a “multiple addition” functional definition for product whose result is the same type, length, as its two input arguments. This can be used recursively on all the rods making up a tower.

When we ignore the categorical structure of the student's work and emphasize instead its arithmetic nature they experience (become “aware of”) mixed number (rational) arithmetic. In this way concrete objects support an experience of number systems. The term “cryptomorphism” describes a non obvious equivalence between number systems and sets of rod constructions. We model this equivalence in the functional programming language Haskell (Benson and Cane, 2017): objects as types and morphisms as functions (Fong et al., 2020). A Haskell “type signature” lists the types of a function's arguments and result.

Our revised model for commutativity of product required a function with type signature (length , length) -> length, rather than (length, length )-> area. Using Haskell as a category is a productive way of thinking about the transformations between the operations of Cuisenaire rod and pattern construction, structured diagrams and written algebraic and arithmetic expressions and equations. The online Appendix to this article contains an annotated version of the flow diagram in Benson et al. (2022) that illustrates the Cui curriculum progression in which students learn to manipulate polynomial representations of numbers in different bases.

## 2. Materials and methods

### 2.1. A co-design framework for appraising equational reasoning

In keeping with Gattegno's rubric of the “subordination of teaching to learning,” and to maximize fidelity to Cui, we developed a co-design framework with teachers for formative assessment of students' equational reasoning. Voogt et al. (2016) report that collaborative design has a positive affect both on professional development and on the implementation of curriculum change, since teachers develop competencies and practice and develop ownership of the change. In drawing on the design of the Berkeley study we asked whether Brownell's findings might be reproduced over the first four terms of schooling, and if so whether they persisted over the subsequent two terms in which Cui was replaced by traditional instruction directed toward proficiency in national assessments. We used a battery of academic performance tests drawn from the Stanford Educational Assessment (SEA) collection, in place of the Brownell's Common test and the standard verbal reasoning test (Brownell, 1967b; Project-ILead, 2019). We report how we determined our sample size, all data exclusions, all manipulations, and all measures in the study.

To design our experiment we adapted historical materials—rods and in-service training resources—to a modern classroom research setting with virtual rods, iPads, electronic whiteboards and internet connectivity. Spielhagan (2011) and Uncapher (2018) discuss issues of the kind we faced when working at a school and district level to incorporate research based learning technology into classrooms. Guided free play and teacher designed puzzles are an important element of the Cui approach. A recent meta-analysis confirms that guided play has greater positive effect than direct instruction on early maths skills (Skene et al., 2022). The technology enabled us to work with teachers to prepare lessons and record whiteboards as lessons were delivered while closely tracking the progress of learners through workbooks and screen shots. This approach to professional development, through collaborative design in teams, that is specific and linked to the curriculum, has been shown to positively influence teachers' knowledge and practice and impact implementation of curriculum change (Voogt et al., 2016; Benson, 2023). We adapted Zazkis and Herbst (2018) framework for teachers and researchers to collaborate in lesson preparation, puzzles and scripts, professional development, and formative assessment.

### 2.2. Study design

In the present study (*N* = 120) students in treatment and business-as-usual control conditions completed assessments at regular intervals including Brownell's original CUI test of equational reasoning, together with novel tablet-based assessments of arithmetic fluency and relational reasoning. These SEA tests were administered as a set of five-minute modules on an iPad. Six students missed more than one of the four observations, leaving a remaining sample of (*n* = 114) participants. We measure two different aspects for each child: Aptitude and Growth in factual fluency. The early impact of the treatment was well established in meta-analysis. This investigation focuses on the year of growth following the nine-month introduction of Cui in UK Year 1 (aged 5 on entry). We measure factual fluency at four growth points (*g*1..*g*4) and study performance in equational reasoning in the final semester of Year 2.

SEA observations were taken at the end of UK Year 1 (at the end of the third term of schooling, growth point *g*1) and the end of each term of Year 2 (*g*2−*g*4). Our CUI test was administered at the end of the second year. Initially 60 participants were selected from a population of 90 children in each of two schools. Not all children were present at each observation and we imputed missing data when one observation was missing. The resulting data set for the SEA scholastic aptitude and factual fluency analysis consisted of 56 experimental pupils and 58 control, and for the equational reasoning (CUI) analysis consisted of 52 experimental and 56 control.

We adopted a between-schools design as used in studies reported in our systematic analysis (Benson et al., 2022). Brownell took account of a non-experimental treatment and control by matching students to construct a balanced design. In the present study demographic and school quality data suggest that any bias in our findings would be in favor of the control school. As a check on this bias we matched subjects in the treated (X) and control (C) samples according to their relational matching (RM) results at *g*4 using the R MatchIt package.

In the experimental school pupils closely followed Gattegno's Mathematics text-books (GM) for four terms (Gattegno, 1963a,b). Lessons were designed by a teacher with 3 years experience with GM. She worked alongside a newly qualified teacher who taught the parallel second class of 30 children. Half termly meetings were held between the teachers and researchers, at which the lead teacher presented her plans and observations to colleagues in other schools. The study was divided into four phases as shown in Table 1. The intensity of the teaching averaged 40–50 min per day for 12 months. This was similar to the average intensity achieved in Brownell's study (45 min/day). Teachers designed lessons and planned their sequence, innovated tasks and activities, and recorded their lessons as electronic white board files.Their work was cross-referenced to exercises in Gattegno Mathematics and photographs of the learners' rod constructions and written work. Researchers provided a planning template (reproduced in Appendix B), co-designed lessons and observed lesson delivery.

**Table 1:** Study design.

**Phase number**	**Phase name**	**Growth interval**	**X assignment**	**C assignment**	**Duration**
1	Initiation	(□,*g*1)	Cui training	Traditional	9 months
2	Reflection	(*g*1, *g*2)	Cui training	Traditional	3 months
3	Proficiency	(*g*2, *g*3)	Some small programs	Traditional	3 months
4	Follow-up	(*g*3, *g*4)	Traditional	Traditional	3 months

□ indicates no observation.

Teaching in England is moderated in Year 2 by an external review against expectations for children's written work. In addition, schools are assessed between *g*3 and *g*4 by two nationally standardized tests of arithmetic and mathematical reasoning (the Key Stage 1 assessments). For these reasons teaching in the Proficiency Phase in the experimental school moved away from GM. It was replaced by practicing for these proficiency tests and the external review, and with lessons to gain familiarity with conventional diagram notations (such as the abstract number line). The Cui approach was augmented with traditional resources and with computer science lessons. Weekly lesson scripts were created by researchers with teachers for small computer programming assignments in Haskell and systematic lesson observations were carried out including curation and appraisal of logs of each students work.

Several factors distinguished the intervention in Phases 1 and 2 from traditional classrooms:

1. the promotion well-designed mathematical exercises that empower students to reason about equivalence and generalize with little or no mental energy.
2. the challenging, revising and changing of these generalizations by the students making them.
3. the roles of listening, careful attention to the use of language and even silence in learning and teaching.
4. the role of free writing. This allows the student to discover regularities for themselves and gives the teacher the opportunity to see which concepts they have mastered, and elsewhere, where the student is still working at an “empirical” level (Goutard, 2017).

#### 2.2.1. Demographics and school quality

We selected schools for the present study who were rated “good” by the UK Office for Standards in Education (Ofsted). Within this classification schools differ in age profile, levels of deprivation and socio-economic context. The matching between the schools was less than ideal. At *g*3 the mean age of the pupils in the experimental school was 6.1 years (0.258 sd) and in the control school it was 6.23 years (0.258 sd). They also differed in socio-economic context and school quality measures.

In England state schools recruit from their local neighborhood or “catchment” area. For popular schools, such as the schools in this study, these areas can be quite small. This means that we were able to use demographic data collected for the neighborhood as a proxy for parental socio-economic status. The UK government classifies school catchment areas into bands of relative deprivation, using information on income level and the educational level of parents. Using the measure for parental education and skills the experimental school catchment was allocated to the fifth of ten intervals and the control school to the least deprived, tenth, interval. On the income measure the experimental school was allocated to the ninth and the control school to the tenth interval. On the overall multiple deprivation index, taking into account employment, income, health etc. the experimental school catchment area was assigned to the ninth interval and the control to least deprived tenth interval.

The results of the Key Stage 2 (KS2) national assessments at the outset of the study can be taken as a measure of school quality. Five statistics are of note:

1. Overall KS2 Mathematics attainment was 110 Control (C), 106 Experiment (X). 100 is the national average.
2. The % of disadvantaged children taking the test was 4% C 3% X. There is reason to believe that the X figure is understated.
3. The measure of mathematics progress - a value added measure - was 2.2 (C.I. 0.8, 3.7) for C and –0.5 (C.I. –1.9, 0.9) for X. This implies that the C school was in a national cohort four years earlier whose KS1 mathematics attainment was 107.8, whereas the X school at that time was 106.5.
4. The proportion of learners with a high score in reading was 46% C, 33% X.
5. The percentage of learners with medium prior attainment achieving the expected level in mathematics was 98% C, 75% X. That is to say the C-X gap in this measure was 23%.

It might be expected therefore that any bias arising from parental educational level, socio-economic status, age or school quality would be in favor of our control school.

At the end of Year 6 the study sample sat the Key Stage 2 national assessments themselves. Since there had been movement in children either leaving or joining the schools—a result of Covid and families moving into and out of the areas and an intake of children from other countries these Key Stage 2 cohorts were not identical to the students in our study. This particularly affected the control school's performance. The experimental cohort demonstrated resilience throughout the lockdowns and the X school continued to make progress on the fifth measure. As a result the study cohort in their KS2 assessment had eliminated the C-X gap in the percentage of learners achieving at or above expected level at KS2.

### 2.3. Growth point assessments

We used SEA modules for mathematics and reasoning to collect statistics on accuracy and speed of work done. These measures are summarized in Table 2. A number of other observations were also made but are not reported further since they are outside the scope of the present study.

**Table 2:** Assessment tests and measures.

**Test**	**Definition**
*CUI*	A 5 minute paper and pencil post test derived from Brownell (1967b). The subject evaluates ten expressions in all four operations and fractions as operators. Measure is total accuracy.
*AF*	Arithmetic fluency. A 3 minute test evaluating as many expressions as possible involving either single digit addition summing to 9 or subtraction of a single digit from a number less than or equal to 10. Conducted on an iPad. Measures are trial accuracy and response time.
*RM*	Relational matching. Subjects are asked to indicate whether two pairs of objects which can differ in 2 properties are alike in the same way. There are 23 trials. Conducted on an iPad. Measure is total accuracy.

The AF and RM modules were administered at each growth point. At *g*4 the module CUI was also assessed.

**Figure 1:**
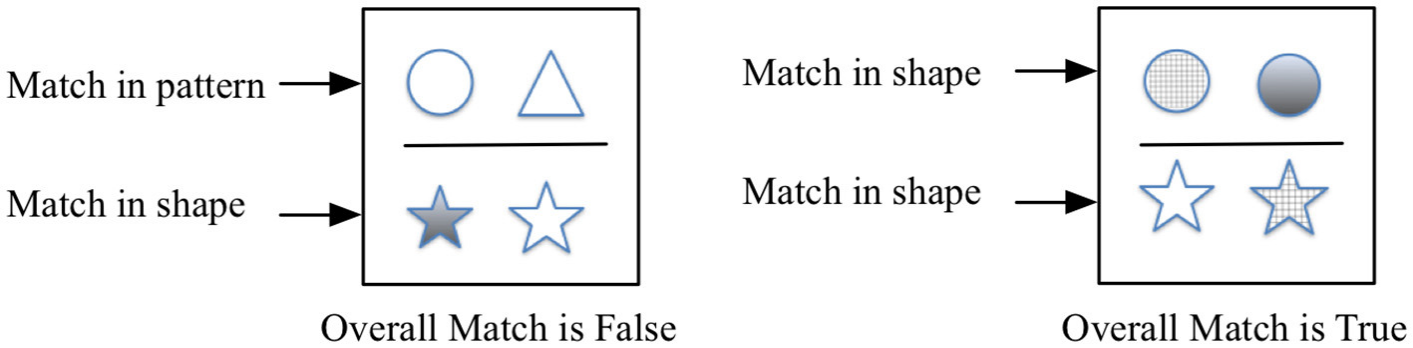
Relational matching (RM) logical reasoning test (Starr et al., 2022).

Brownell found it was important to test for differences in general scholastic aptitude outside the domain of mathematics and used a verbal reasoning test. Meta-analyses of the impact of manipulatives on mathematics learning expect that older students who have developed the ability to reason abstractly will benefit most from instruction that consists exclusively of symbolic representations (Carbonneau et al., 2013). Increasingly executive function tests such as working memory or task switching and tests of global fluid intelligence are being used to assess domain-independent reasoning skills. Our choice of Relational Matching (RM) is motivated by executive function approaches as a non-verbal reasoning test for scholastic aptitude that can capture the aspect of general reasoning while de-emphasizing potential language differences between subjects (Christoff et al., 2003; Starr et al., 2022). RM is designed to test similar operations to tests in the Raven's Progressive Matrix family. The more complicated Raven's tests, closer to problems found in standard scholastic aptitude tests, require analysis as well as visuo-spatial skills. SEA RM is such a logic test. It requires two steps of analysis (i) to determine how a pair of objects which are characterized by two properties (shape and fill pattern) differ from one another and (ii) to distinguish whether two such pairs differ in the same way (“True”) or not (“False”) (Figure 1). There were 23 trials at each growth point. A high score is indicative of a good performance.

The CUI test enabled us to explore a second aspect of the equational reasoning intervention in addition to the arithmetic proficiency tests and accuracy in solving complex problems. Brownell set these questions at the end of Primary Year III, a year later than the present study. We did not expect therefore to find the degree of accuracy that he reported. We were able however to use his test items to assess the disposition of the learners to persist in an unfamiliar problem space—sometimes called “growth mindset”.

**Figure 2:**
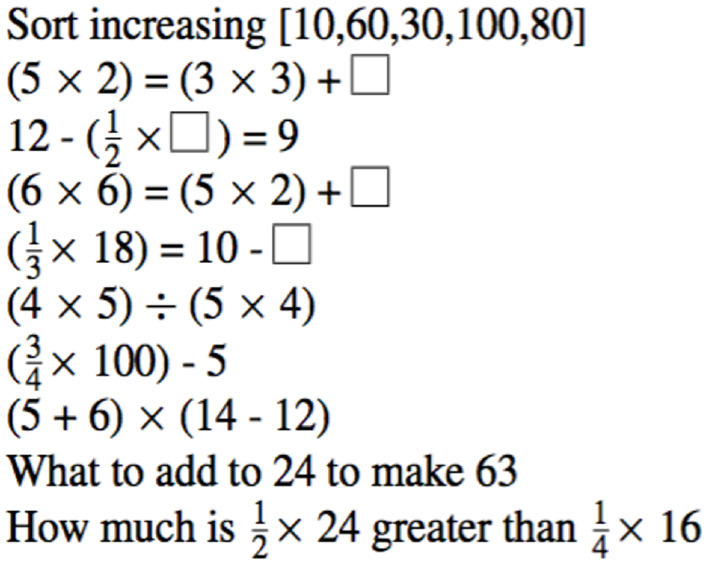
CUI test items (*g*4).

CUI was a 15-min pen and pencil test in two parts. During the first 10 min practice session the invigilator led the class in a discussion about how they might approach 20 questions drawn from Brownell's CUI test protocol (Brownell, 1967b, p. 250). Attention was drawn to the multi-step nature of these calculations. The control school was not familiar with this kind of questions. The discussion was a way to familiarize them with what was required, while it refreshed the experimental subjects' experience with these questions. Ten further questions from Brownell's CUI test were posed in written form as set out in Figure 2 and completed in silence. In this way CUI assessed the degree to which traditional learning in the control school prepares pupils for equational reasoning. For the experimental school, whose recent experience was with single step questions as required by the national assessments, CUI assessed the extent to which they had retained and had confidence to build on what they had learnt in Phases 1 and 2.

## 3. Results of the longitudinal study

We conducted an analysis to compare the arithmetic fluency accuracy and response time and relational matching accuracy of the two groups. We found that fluency in equational reasoning, gained through fidelity to the Cui curriculum, led to gains in arithmetic fluency in the six months following the Cui intervention. Based on a meta-analysis of pre-post study effects showing a mean effect size of 0.66 (Cohen's d), we determined that our school partner sample size—nX = 56, nC = 58—would enable us to detect this mean effect size with at least 80% power assuming a 2 sample T-test was used with null hypothesis (H0): mean X = mean C and rejecting H0 if *p* < 0.05 (Benson et al., 2022). The actual power was 93% for d = 0.66 and 74% for d = 0.5.

The study found as expected that there was a correlation between relational matching skill and mathematics performance. However when this was controlled for in a regression model it could not account for all the gains in performance in arithmetic fluency achieved by the experimental group. We put the experimental and control subjects into the same sample space by matching the subjects on the basis of their performance in a relational matching task. We matched the subjects in the two samples according to their RM measure and found that there was an interaction between treatment and domain general reasoning skill. We found that fluency in ER, gained through fidelity to the Cui curriculum, led to gains in arithmetic fluency in the six months following the Cui intervention.

### 3.1. Accuracy and response time

Tables 3, 4 show the difference of the means for AF and RM observations between the X and C samples for each term together with the 99.5% confidence intervals that there is a significant difference between the means. It shows that there is a significant C-X difference between the mean RM scores at *g*3 and *g*4. Otherwise the results are comparable.

**Table 3:** Summary statistics and C.I. for difference in means (C-X) AF.

**Growth**	**X sample (*****n*** **= 56)**		**C sample (*****n*** **= 58)**		**99.5% C-X**	
**Point (GP)**	**Mean**	**SD**	**Mean**	**SD**	**Confidence**	**Interval**
g1	20.2	8.63	21.7	8.48	–1.68	4.68
g2	25.1	8.44	26.8	8.35	–1.42	4.82
g3	31.4	7.90	30.7	9.44	–3.94	2.54
g4	32.9	9.42	32.1	8.62	–4.15	2.55

**Table 4:** Summary statistics and C.I. for difference in means (C-X) RM.

**Growth**	**X sample (*****n*** **= 56)**		**C sample (*****n*** **= 58)**		**99.5% C-X**		
**Point (GP)**	**Mean**	**SD**	**Mean**	**SD**	**Confidence**	**Interval**	
g1	12.2	3.24 13.1	3.68	–0.39	2.19	
g2	13.3	2.94	14.1	3.59	–0.42	2.02	
g3	12.5	2.97	14.9	3.76	1.14	3.66	^***^
g4	14.4	4.17	16.8	3.80	0.92	3.88	^**^

Significance codes 0“***” 0.001“**” 0.01“*” 0.05“.” 0.1“;”.

**Figure 3:**
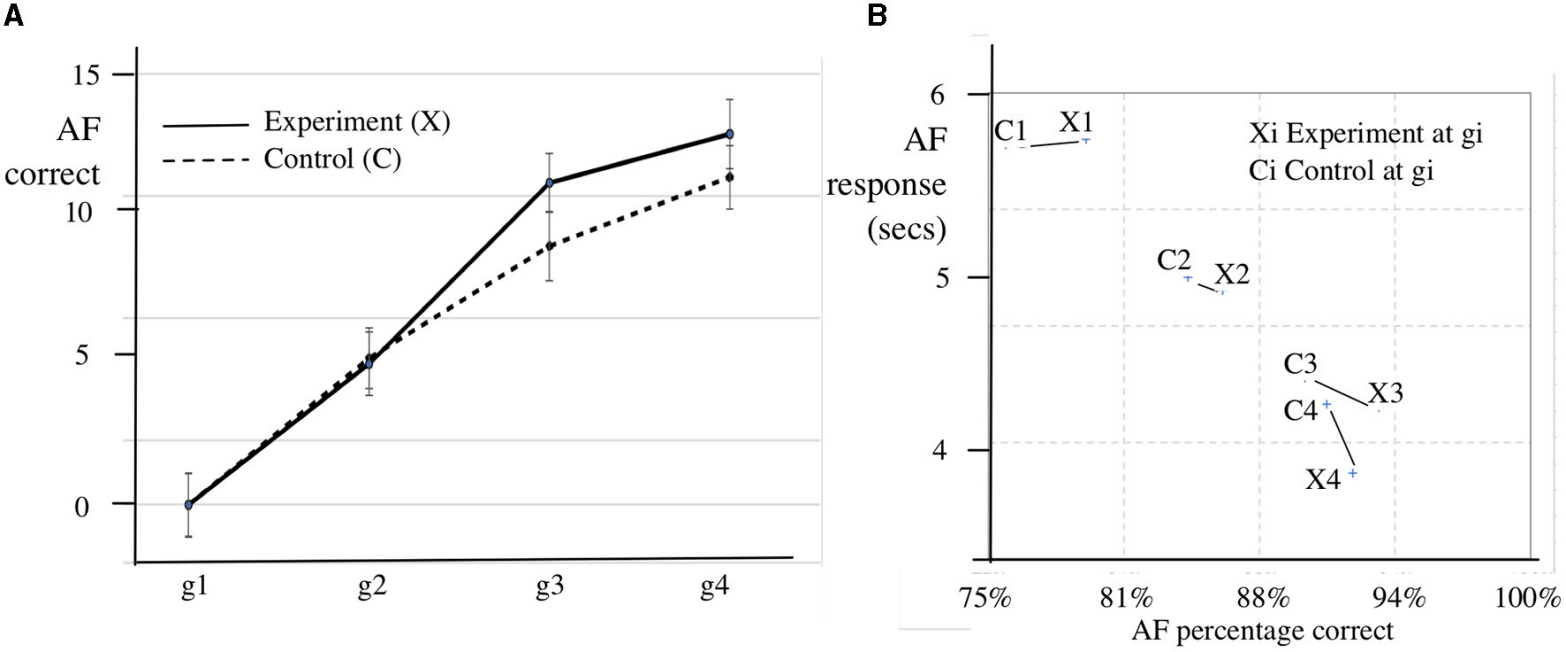
SEA AF mean accuracy gains, mean proportion correct and mean response time by growth point. **(A)** AF mean accuracy gains by growth point. **(B)** AF mean response time (secs) by proportion correct.

Figure 3A shows the mean increment in AF accuracy for the experimental and the control groups by term over the course of the study. Figure 3B shows the relationship between the response time and proportion correct. In general the faster response is related to increased accuracy, but there appears to be an upper limit to this process beyond which increased speed impairs accuracy.

Figure 3 shows the intervention (*g*1, *g*2) prepares pupils for future learning, in that gains in speed and accuracy relative to the control group appear in Phase 3 (*g*2, *g*3) and the Follow-up Phase (*g*3, *g*4), after the Cui intervention has ended.

#### 3.1.1. Scholastic aptitude

**Figure 4:**
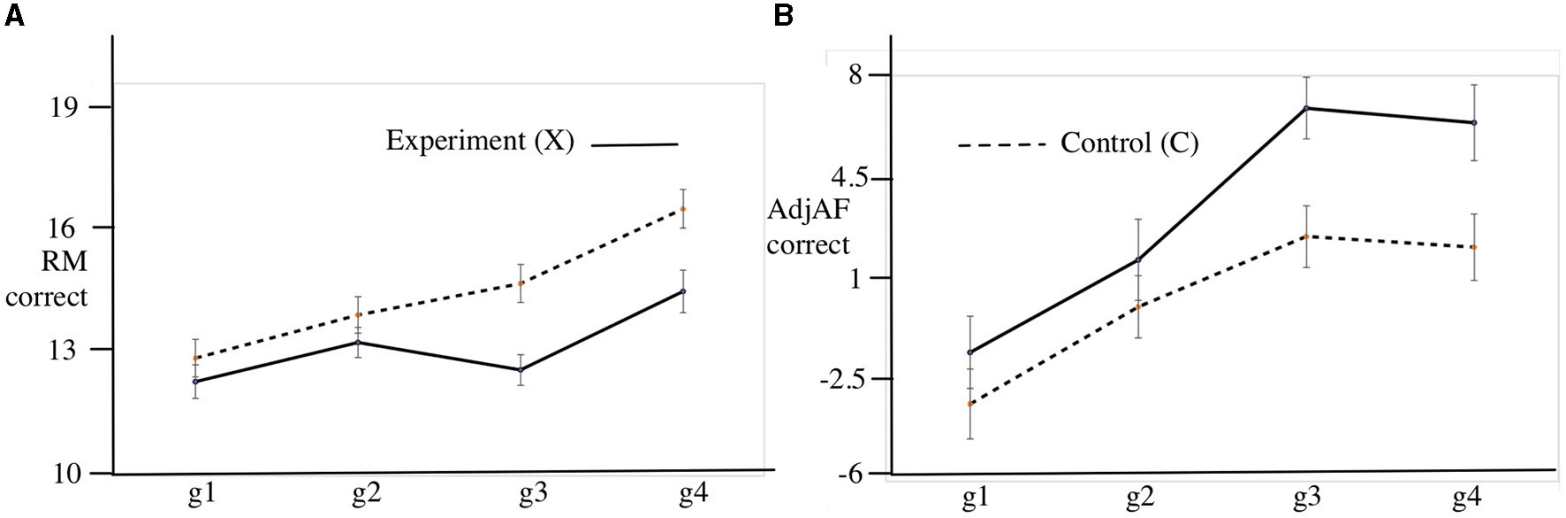
RM and efficiency of learning (adjAF) by sample and growth point with Standard Error bars. **(A)** Relational matching (RM) mean accuracy. **(B)** Mean correct AF attempts exceeding prediction based on RM (AdjAF).

Figure 4A shows the mean RM accuracy for the experimental and the control groups by growth point. There is a sustained gap in relational matching skill in favor of the C sample as measured by this executive function test.

#### 3.1.2. Growth in efficiency of learning

We propose a measure *adjAF* (adjusted arithmetic fluency) of the efficiency of learning. This quantifies the extent to which the AF accuracy for a subject exceeds a prediction based on their RM measure at each growth point. It is calculated by first averaging AF and RM over all four growth points (GP) for each subject. We then built a linear regression model *avAF*~*avRM* and extracted the intercept (8.0323) and slope (1.4059). These coefficients were used to predict a value for AF for each subject at each growth point using the formula *predAF* = 8.0323+1.4059*RM. The adjusted AF accuracy was computed as *adjAF* = (*actual*)*AF*−*predAF*. We then investigated the model *adjAF*~Treatment*GP and found that Treatment gave rise to statistically significant excess in AF over that predicted from RM alone. The ANOVA summary is shown in Table 5.

**Table 5:** ANOVA for linear model for adjAF predicted by treatment and growth point (GP) *n* = 114.

	**Df**	**Sum Sq**	**Mean Sq**	**F value**	**Pr(>F)**	
Treatment	1	404	403.51	5.6992	0.01739	^*^
GP	3	5076	1691.88	23.8962	2.311e-14	^***^
Treatment:GP	3	608	202.75	2.8636	0.03642	^*^
Residuals	448	31719	70.80			

Significance codes 0“***” 0.001“**” 0.01“*” 0.05“.” 0.1“;”.

Figure 4B shows the increment in mean *adjAF* accuracy for the experimental and the control groups by growth point. The acceleration in *adjAF* between *g*2 and *g*4 for the X sample in comparison with the C sample is consistent with a delayed ‘sleeper effect' from the intervention, which ceased at *g*2. *adjAF* has a Cohen's d at *g*4 of 0.497, a medium effect size.

### 3.2. Equational reasoning

**Figure 5:**
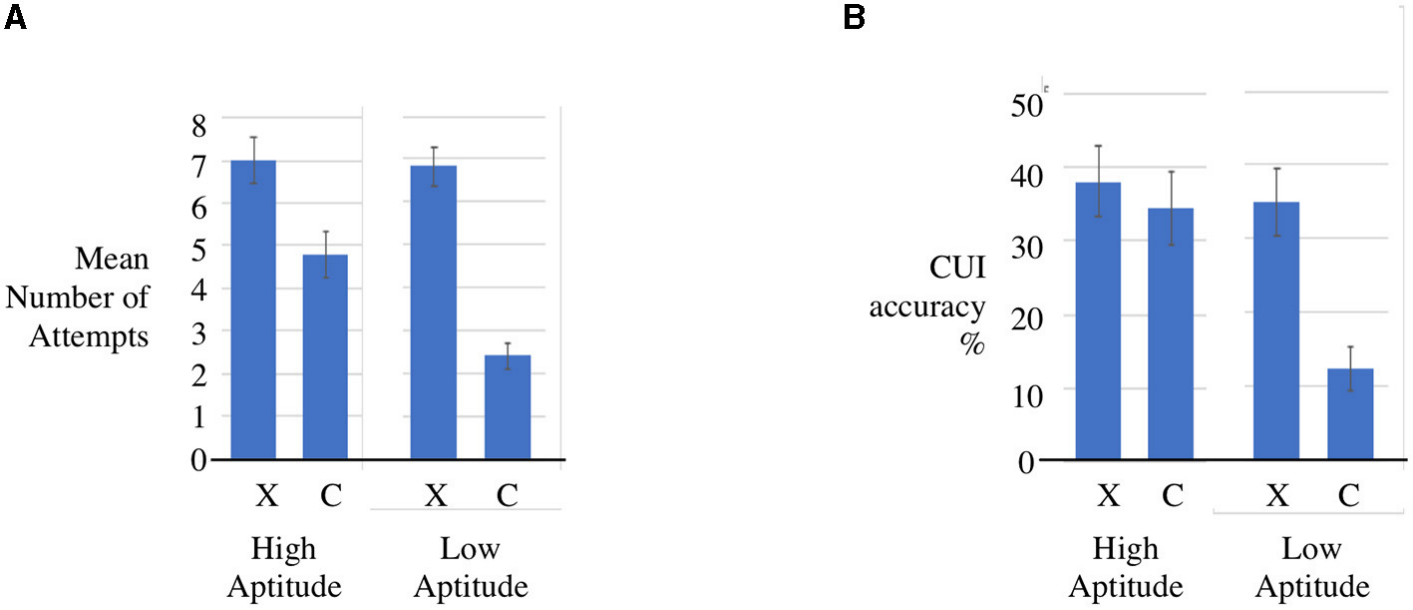
CUI interaction effects by aptitude and sample **(A)** disposition to solve problems and **(B)** accuracy (g4). **(A)** Disposition to attempt CUI by scholastic aptitude (High, Low) and sample [Experiment (X), Control (C)] with standard error bars. **(B)** Mean CUI accuracy % by scholastic aptitude (High, Low) and sample [Experiment (X), Control (C)] with standard error bars.

We explored two aspects of the equational reasoning intervention: one was the disposition to persist in an unfamiliar problem space—sometimes called ‘growth mindset' – and the other was fluency in reaching a correct solution. We also found that Cui training had a significant impact on disposition. Figure 5A shows the disposition to attempt to solve problems of students with experience of Cui compared to the control group. That is, the experimental group showed a greater willingness to engage with challenging problems instead of skipping them. We permitted skipping as an option as we wanted to test meta-awareness - the degree to which the students could estimate their own competence. Here we found no contrast: both groups had the same success rate, as measured by the ratio of correct to incorrect responses.

Figure 5B shows the aptitude-treatment interaction for CUI mean accuracy with the samples matched as discussed in Section 3.3. The raw data is reproduced in the online Appendix. Because students were allowed to skip questions we do not know what the performance of the groups would have been if they had been forced to answer. This may have skewed the results in favor of the experimental group since they attempted more questions. In fact the mean CUI correct score for the X sample was 3.52 (sd 2.37) and for the C sample was 2.57 (sd 2.61).We conducted a Welch two-sample t-test comparing these statistics which confirmed that the difference between the means was likely to be significant. The Cohen's d effect size was 1, which is a large effect size.

We asked how CUI performance in equational reasoning was predicted by the growth in AF accuracy. We found that using *g*1 and *g*3 was best with the model generating similar coefficients. The ANOVA is shown in Table 6. It shows that growth at *g*1 is significant at the p < 0.001 level and growth at *g*3 at the *p* < 0.05 level.

**Table 6:** ANOVA for linear model for CUI predicted by adjusted AF showing relationship between treatment effect on AF at *g*1 and *g*3 and performance in the CUI test at *g*4.

	**Df**	**Sum Sq**	**Mean Sq**	**F value**	**Pr(>F)**	
adjAF1	1	96.81	96.811	18.2482	4.272e-05	^***^
adjAF3	1	31.06	31.059	5.8544	0.01726	^*^
Residuals	105	557.05	5.305			

Significance codes 0“***” 0.001“**” 0.01“*” 0.05“.” 0.1“;”.

We interpreted the coefficients as +0.15 for *adjAF*1 and +0.08 for (*adjAF*3−*adjAF*1). This suggested that where a subject ended Year 1 is the main effect but subsequent growth (after allowing for RM) between *g*1 and *g*3 was also predictive of CUI. In other words there are substantial benefits in laying the foundation for equational reasoning in Phase 1. These are reinforced during the Phase 3 and the Follow-up phase when students were preparing for a common standardized assessment and teacher appraisal.

### 3.3. Balanced appraisal

**Figure 6:**
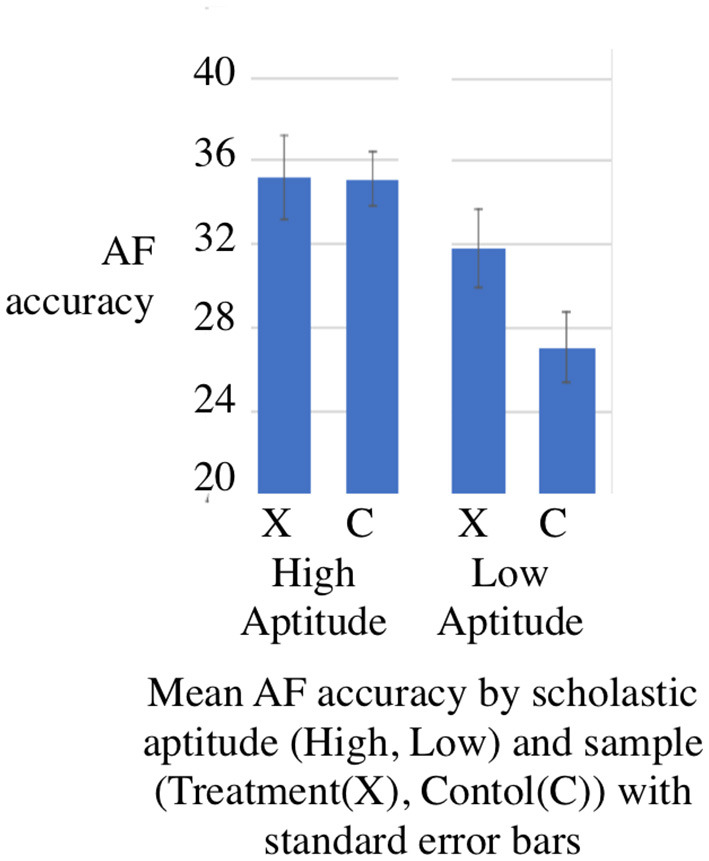
Aptitude-treatment interaction effects AF accuracy present study (matched data) at *g*4.

As a check on our analysis, and any bias introduced by the non-experimental design, we constructed a matched model using the R package MatchIt. This was used to divide the experimental sample into two same size sub-samples–labeled (H)igh SA and (L)ow SA using the non-verbal reasoning task RM at *g*4 as a proxy to correspond to Brownell's scholastic aptitude (SA) verbal reasoning test which was undertaken at the end of his study. The experimental sample was divided by Matchit into High and Low SA in the ratio 2:3, the control sample in the ration 3:2. Matchit created weights for input to the R core function lm to check how AF accuracy is related to the Growth Point, Treatment and SA using the formula *AF* ~ GP + SA + Treatment*SA. The ANOVA shown in Table 7 shows a Treatment:SA interaction with *p* < 0.01. This is statistically significant. Figure 6 shows the mean accuracy in the common tests by subsample in this weighted model. These results are consistent with Brownell's finding of a treatment-interaction in his high intensity studies (Brownell, 1967b, p. 49). As a check that this effect was not evident at the outset we ran the model using the dataset for AF at *g*1 and found no significant interaction.

**Table 7:** ANOVA for linear model for AF predicted by Growth Point (GP), Treatment and Scholastic Aptitude (SA).

	**Df**	**Sum Sq**	**Mean Sq**	**F value**	**Pr(>F)**	
GP	3	8234.0	2744.68	41.1619	<2.2e-16	^***^
SA	1	2988.3	2988.33	44.8159	7.008e-11	^***^
Treatment	1	373.3	373.28	5.5981	0.018436	^*^
Treatment:SA	1	638.2	638.23	9.5715	0.002109	^**^
Residuals	417	27805.6	66.68			

Significance codes 0“***” 0.001“**” 0.01“*” 0.05“.” 0.1“;”.

We also used the weighted model to check how CUI accuracy is related to Treatment and scholastic aptitude (SA) using the formula *CUI* ~Treatment*SA. The ANOVA shown in Table 8 shows a Cui treatment effect in favor of the experimental sample (F factor 10.9994, *p* = 0.001256 < 0.01) which is statistically significant. We also carried out a 2-sample t-test *CUI* ~ *Treatment* comparing high SA and low SA groups separately. The Bonferroni corrected Welch tests for both High SA (*p* = 0.01 < 0.25) and Low SA (*p* = 8.134e-0.09 < 0.25) were statistically significant. Finally an analysis of Cohen's d showed a large effect size (d = 1.18) for the Low SA sample and a small effect size (0.18) for the High SA sample. The ratio of 6.5:1 (1.18:0.18) in the comparison of the two Cohen's d effect sizes confirmed a more substantial effect for the Low SA group.

**Table 8:** ANOVA for linear model for CUI predicted by Treatment and SA.

	**Df**	**Sum Sq**	**Mean Sq**	**F value**	**Pr(>F)**	
Treatment	1	57.69	57.693	10.9994	0.001256	^**^
SA	2	31.41	115.707	2.9946	0.054391	.
Residuals	104	545.49	5.245			

Significance codes 0“***” 0.001“**” 0.01“*” 0.05“.” 0.1“;”.

The ANOVA, *t*-test and effect size evidence together warrant our central claim that treatment interaction was significant and also that the effects are strongest for children demonstrating lower scholastic aptitude.

## 4. Discussion

### 4.1. Findings

In this paper we have explored Brownell's findings and hypothesis that the equational reasoning (ER) skill gained by following the Cui approach underpins later arithmetic and algebraic proficiency. We investigated two aspects of his intervention: one was the disposition to attempt multi-step questions, and the other was fluency in reaching a correct solution. We replicated his study of the efficacy of the Cui approach using the Stanford Educational Assessment tool (SEA). The longitudinal transfer study was really important because the gain in arithmetic proficiency is bigger than the effect of Cui training at the time. That is, Cui training is having a bigger effect on learning after the training is completed. Not only did we replicate Brownell's findings for proficiency but like him we found that learners were more daring in their approach to complex problems. We report a treatment interaction effect for arithmetic fluency and for CUI disposition, and an interaction effect for CUI accuracy that could reflect both.

The systematic review reported in Benson et al. (2022) suggested that fidelity of transmission is a moderator in arithmetic proficiency. The lead teacher in the present study had three years prior experience with the approach. She was able to integrate Gattegno's program as set out in the online Appendix into the school's medium-term lesson plans for Key Stage 1. This was comparable to the average teaching intensity in Brownell. Our findings reproduce Brownell's main conclusions. In particular they support his hypothesis that “prior attention to the conceptual aspects of arithmetic (will) pay large dividends in increased proficiency in the end and there is reason to believe that, if proficiency were stressed later on, the hypothesis would be established” (Brownell, 1967a, p. 117) .

In replicating the earlier experiment design we used modern psychometric and statistical techniques to study learning and scholastic aptitude. Schwartz et al. (2005) propose an expanded definition of transfer of learning to encompass an enriched notion of education. They contrast assessments of “preparation for future learning” (PFL) with the sequential problem solving encountered in standardized assessments. Their studies suggest that early innovation leads to better adaption to new challenges in the short run and better efficiency in the long run in transfer situations. We have followed their suggestion that PFL is assessed through two independent measures of *efficiency* and *innovation*.

There was a difference in scholastic aptitude between the two schools as measured with the SEA RM test which we attribute to different demographics. The *p*-value was less than 0.001 at *g*3 and less than 0.01 at *g*4. These effects are statistically significant. We took SEA measures of RM to be equivalent to Brownell's verbal reasoning measure of scholastic aptitude (SA). We divided the samples into two groups SA High or Low by matching and found a two-way interaction between Treatment and SA in predicting arithmetic fluency (AF). This interaction had a *p*-values of *p* < 0.05 which is statistically significant. The magnitude and direction of the effects for our study were similar to Brownell's.

We proposed a measure for *efficiency in future learning* as the increment in growth in AF accuracy not predicted by SA. We found a medium effect size (d = 0.457) in favor of the school that received the Cui treatment at the end of Year 2. Since the Cui intervention terminated six months earlier this was a measure of the degree to which Cui prepared pupils for efficiency in arithmetic computation in the national assessments. We observed a sleeper effect in that gains in efficiency accelerated after the conclusion of the intervention.

In addition to the benefits of the Cui intervention in the AF score the experimental students showed a greater disposition to engage with multi-step equational reasoning in the CUI test. Giving students the option to skip questions means that we must be cautious in interpreting the overall accuracy results. Nevertheless we report an educationally significant difference in performance and a large effect size (d = 1.0) in favor of the experimental school in this test. The CUI measure was devised by Brownell. We use it to assess the impact of the *innovation* on future learning and teaching.

### 4.2. A categorical account for the Cui sleeper effect

Sleeper effects are seldom reported in the literature, and they have not in general been studied in relation to transfer and preparation for future learning. The present study's Cui sleeper effect, evidence for Brownell's treatment interaction, presents an opportunity to relate pedagogy to our growing understanding of the neurophysiological basis of mathematical understanding.

Vandell et al. (2010) describe how the benefits of pre-school programs boost later academic performance. Barrera et al. (2002) in a study of preventative interventions on aggression highlighted the importance of measuring long term effects. Bailey et al. (2017) review the nature of interventions that lead to persistence or even later emerging effects compared to those that quickly fade out. They identify three distinct processes that might account for these effects: skill building, foot-in-the-door capacity building, and sustaining environments. All three are evidenced in the Cui intervention: learners gain skill in multi-step expression evaluation, they are introduced from the outset to reasoning about equivalence which is an essential underpinning of school mathematics and their first encounters with mathematics are in creative and playful environments, which encourage a highly abstract and systematic form of learning.

Cui teachers educate learners' sensitivity to common patterns of mathematical relations by coordinating sight, hearing, touch, fine motion (writing, drawing, and construction) and introspection. The integers are introduced to teachers as the names for a sequence of patterns constructed by partitioning rods. The sequence exhibits a kind of “perceptual productivity,” by using combinatorial and recursive functions to construct limitless complex diagrams (Barsalou, 1999; p. 592, Benson, 2015). Our categorical account postulates that “Cui training” activates and reinforces a more extensive brain sub-network than instrumental drill and practice—-one that privileges reasoning about equivalence and verification over memorisation and recall (ATM, 2018). It proceeds through three stages as a preparation for future learning.

In Phase 1 of the present study experimental students are given an opportunity to experience the algebraic structure of the number system by constructing complete patterns and through free writing of equivalent expressions in all four operations and fractions as operators. The intervention strengthens their awareness of the structure of the whole number system which enhances subsequent factual fluency when efficiency is emphasized.

Phase 2 consists of exercises that employ what has been learned about addition facts to 10 and extends their Cui knowledge to reason about multiplicative relationships, factors and division through new rod constructions - crosses and towers.

Phase 3 and the Follow-up Phase are occasions for future learning to meet the common (external) requirements of the arithmetic and reasoning standardized tests and teacher moderation. The experimental sample is distinguished from the control sample by their familiarity with reasoning about the equivalent value of integer expressions. We also found that they were more willing to engage with challenging multi-step CUI problems. While they had encountered such expressions in Phase 1, this facility was not required in their practicing for the national assessments.

### 4.3. Limitations

A central limitation of this study is consistent with limitations of other quasi-experimental designs in that they do not involve randomization at the class or student level. Although our design was based on high fidelity replication of Brownell's, our methods departed in several noteworthy ways.

His Common arithmetic test was designed by teachers to cover material present in both X and C schools. We replaced this with an arithmetic facts fluency test based on the Woodcock-Johnson Mathematics Fluency subscale (Woodcock et al., 2007). Fluency with single digit arithmetic is a standard schools are expected to reach by *g*1 yet performance on this metric continues to grow well beyond this point. Brownell's CUI test examined material that was covered by the X schools, but not the C schools. We adapted his test to reflect the shorter, 2-year, duration of our study. We did not replicate his TRA test of material covered by the C schools but not the X schools.

We did not match the treatment and control samples in the same way as Brownell. He recruited students from 24 schools and created a balanced quasi-experiment by matching their scholastic aptitude obtained with a standard verbal reasoning test, removing the middle 20% from the distribution and mimicking a balanced experimental design (Benson et al., 2022). We performed a statistical analysis using the R MatchIt library to match subjects according to their RM observations. This resulted in similar findings to those reported by Brownell although our sample size was too small to produce his aptitude-treatment interaction in the case of the CUI test.

### 4.4. Conclusions

The present study has highlighted that Cuisenaire rods can have a large effect on arithmetic proficiency, equational reasoning and transfer of learning if rigorous attention is given to the appropriate curriculum and pedagogy. We found that codified lesson scripting coupled with technology, enhances planning and communication between researchers, teachers and learners and increases fidelity. Although the paper falls short of a randomized controlled trial in a field not already rich in empirically motivated RCT, we need more studies like this to motivate future work.

## Data availability statement

The original contributions presented in the study are included in the article/Supplementary material, further inquiries can be directed to the corresponding author.

## Ethics statement

Ethical review and approval was not required for the study on human participants in accordance with the local legislation and institutional requirements. Written informed consent to participate in this study was provided by the participants' legal guardian/next of kin.

## Author contributions

IB and BM contributed to conception and design of the experiment. IB organized the dataset. IB, NM, and BM performed the statistical analysis. IB wrote the first draft of the manuscript. NM and BM wrote sections of the manuscript. All authors contributed to manuscript revision, read, and approved the submitted version.
