# Supplementary material for: Interventions to improve equational reasoning: replication and extension of the Cuisenaire-Gattegno curriculum effect
Source: Front Psychol. 2023 Aug 28;14:1116555. doi: 10.3389/fpsyg.2023.1116555 (PMC10509469; doi:10.3389/fpsyg.2023.1116555)
Supplement: Supplementary file 1 [file Data_Sheet_1.PDF]

Interventions to Improve Equational Reasoning: Replication and extension of the Cuisenaire-Gattegno curriculum effect. *Supplementary material*

Introduction

In this appendix we describe the design of a series of 80 lesson units that formed the basis for our intervention and the percentage cumulative accuracy, skip and failed attempts for the 10 question CUI test.

1. Curriculum progression

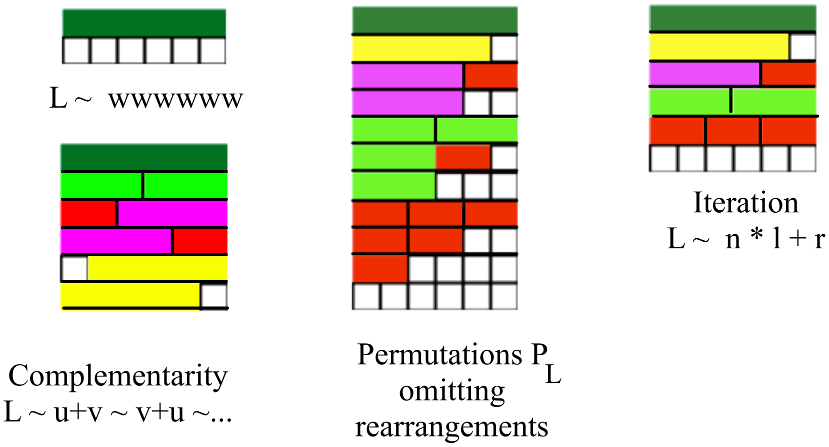

Figure 1: *Gateway nodes in the Curriculum Graph (Cane, 2017, p. 8)*

Gattegno uses operations with the rods – placing them end to end, side by side or stacked as crosses or towers – to model sets with structure such as the integer and rational number systems (Choquet, 1963, p. 4). He created a directed graph of the logical dependencies between mathematical concepts developed from the study of the table of partitions of the rods. It has four root nodes we call 'gateway patterns'. These nodes are illustrated in Figure 1 with a set of patterns for the dark green (*d*) rod. Gattegno calls the web of concepts a 'temporal hierarchy' (Gattegno, 2010a, p. 193). The graph shows how concepts depend on one another, and helps the teacher to plan lesson sequences. One route through the graph is shown in the annotated ATM chart (Figure 2).

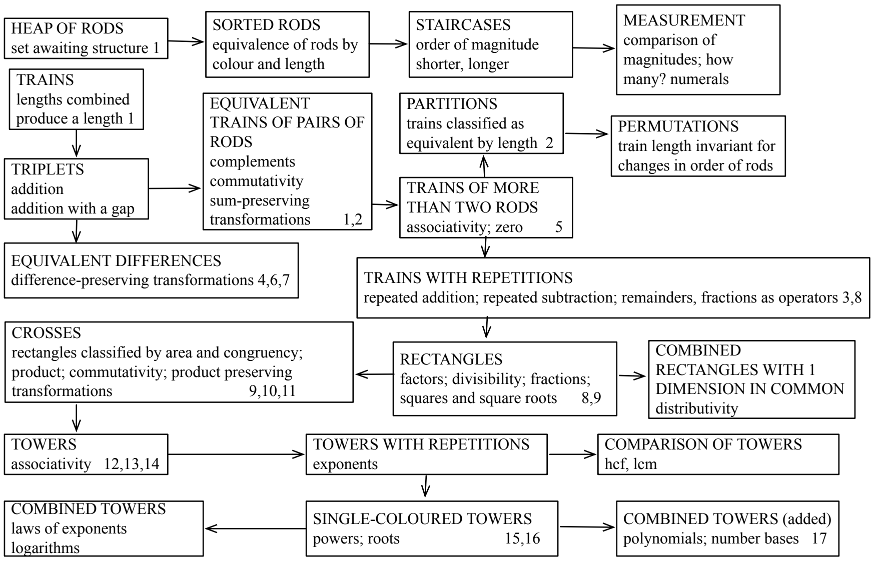

Figure 2: *An annotated flow chart for the Cui approach to coordinating vision, audition, haptic, sensorimotor and introspective modalities (ATM, 1977, p. 185)*

There were three key lesson planning documents: the ATM chart, Gattegno's curriculum graph (Gattegno, 2010a, p. 193), and a medium term plan. The ATM and Gattegno diagrams show logical dependencies between elementary mathematical concepts developed from the study of the table of partitions of the rods. The medium term plan in Table 1-2 is a sequence of 80 units taken from the early chapters of Goutard (2017) together with activities selected for a study of the Cui approach carried out by Sweeney for the Kitchener School Board (Sweeney, 1964). They are cross referenced to chapters and exercises in GM. Detailed design of each lesson varied as the teachers learnt from their students' reactions, and the order in which discoveries spontaneously occurred. In the experimental school planning was led by an experienced teacher, working alongside a newly qualified colleague. Two classes of 30 pupils took part. In the control school three classes were involved. The school selected a representative 20 pupils from each class to take part in the study.

The first steps in the Cui programme are to co-ordinate the relationships of equivalence and order by arranging rods so that they form a progression Gattegno calls a *staircase*: e.g. $w < g < y < k$...... These writings name the process of making constructions with the rods and they record the relationships between their lengths. Learners move fluently between these representations of mathematical ideas so that the elements and actions of one structure (the rod world) can be substituted for the elements and operations of another (written expressions): a 'cryptomorphism.'

The intervention starts by sorting the rods according to their colour, and again according to their length. Students find that rods are either the same colour and length, or they are distinct colours and lengths. These relationships of *equivalence* and *order* are signified using symbols and letter codes.

From the earliest lessons the concepts and symbols =, +, –, $>$, $<$, $\times$, $\div$, $\sim$ are explained in an algebraic context that students can use and understand. For example, we may observe that $y \sim y$ and $k > r$. That is, by holding two rods side by side and gesturing we say "This y is *equivalent in length* to that y" or "k is longer (bigger) than r." We created structured diagrams ('ideograms' Figure 1) by making images of the rods juxtaposed and aligned at one end on an interactive white board, and give the diagrams symbolic names: $y \sim y$ and $k > r$.

In sessions of free play, and free writing, the students become aware that they can move freely between: imagining an activity of putting rods end to end (to make a *train*) and side by side (a *difference*), recognising that there are many ways to choose a pair of colours to combine, simultaneously perceiving a train and its component cars, and understanding that the written sum is both inherent in the train and distinct from it.

With the discovery of *addition* (putting the rods end to end) it is possible to compare trains of rods: $k+w < y+p$, $y \sim r+g$.[1]

Next 'tables' are formed by partitioning a given length (known as a 'family of equivalent sums')[2]:

$p \sim g+w \sim w+g \sim r+r \sim r+w+w \sim ...$

The orange rod is longer than the yellow, and it is also longer than the red. Is it a little longer, or a lot? When Goutard comes to establish these connections in a precise way, *whole numbers* and *fractions* appear naturally. For example, the orange rod is equivalent to two yellows or five reds. The other way round we say the yellow is equivalent to "half of" the orange and the red to "one fifth."[3]

From the 'tables', we get *subtraction* as another way of reading:[4]

$y \sim r+?$ $\quad$ $y-r \sim ?$

The latter equation is read as "what is the *difference* between yellow and red?" There are eight ways of writing this relationship:

$y \sim r+g$ $\quad$ $y \sim g+r$ $\quad$ $y-r \sim g$ $\quad$ $y-g \sim r$

$r+g \sim y$ $\quad$ $g+r \sim y$ $\quad$ $g \sim y-r$, $\quad$ $r \sim y-g$

If we measure each rod with the white rod we find that each length can be made with an all white train.[5] The number of whites we need gives a *numerical synonym* for each rod. (We can of course obtain other numerical names by measuring with a different colour). We use these names to map diagram names (*equations*[6]) from their letter form into 'equalities' where 'equivalent length' becomes 'equals'. In this way we arrive at 'families of equivalent differences:'[7]

5 = 6 - 1 = 7 - 2 = 7 - (1 + 1) = ... = 17 - 12 = ...

Certain tables of partition have the special property of containing only rows formed

[1]The gateway node *Complementarity*, which is connected in the curriculum graph by arrows to the *commutativity* and *associativity* of *addition*

[2]Permutations P_L omitting rearrangements

[3]The gateway node *Iteration*

[4]Subtraction

[5]The gateway node $L \sim ww...w$

[6]Equations

[7]Classes of equivalent differences

of rods of the same color: [8] e.g.,

$d \sim g + g \sim r + r + r \sim w + w + w + w + w$

We call this the 'table of factors' for a given length.

The rods of each of these rows may be put side by side to form rectangles (we exclude the case where a square is obtained). The two dimensions of the rectangle give the *factors* of the product.[9] Rectangles that correspond to pairs of factors are congruent. We signify the product by taking one rod from each congruent rectangle and stacking them to form a 'cross.'

Goutard then considers particular decompositions that behave in a particular way: they deserve a particular treatment, and can be written as *multiplications*[10] or the 'family of equivalent products.'

For example, using numerical names for the rods measured with a white rod, the factors for dark green shown as iterations with no remainder in Figure 1 yield:

$6 = 1 \times 6 = 2 \times 3 = 3 \times 2 = 6 \times 1$

Division follows, and is written in various forms ('families of equivalent quotients'):[11]

$6 = 3 \times ?$ $\quad \frac{6}{3} = ?$ $\quad 6 = ? \times 3$ $\quad 6 \div 3 = ?$

Irregularity will soon be found; some numbers have many factors, others few, and some have only themselves and 1. It is therefore useful, when a certain stage of familiarity has been reached with these processes, to try and make an inventory, classifying numbers as *prime*[12] or *composite*.[13] When we are specifically interested in divisibility by 2, we classify into *odd* and *even* numbers.

When examining crosses, it will be noticed that certain products are formed by factors that are prime numbers (2×5), while others contain at least one factor that is a composite number (3×4); that is, one that can itself be broken down into a product. Another cross can legitimately be substituted for one of the rods in the original cross to form a 'tower' (breaking down into a product of prime factors):[14]

$3 \times 4 = 3 \times (2 \times 2)$ $\quad 6 \times 2 = (3 \times 2) \times 2$

Certain crosses and certain towers are distinctive in that they are formed only of rods of the same colour (e.g., $8 = 2 \times 2 \times 2$). We can therefore just indicate the height of the tower in relation to the size of the base (to form an 'L'), and when multiplying two towers of the same color, we add the heights. Goutard again has a special case, and is justified in introducing a new notation:[15]

$8 = 2^3,$ $\quad 8 \times 4 = 2^3 \times 2^2 = 2^5$

As the operation of *raising to a power* is not commutative the two inverse operations can be clearly distinguished and the questions

$8 = ?^3$ $\quad \sqrt[3]{8} = ?$

[8] Single color trains, $L = n * l$ with no remainder

[9] Product

[10] Multiplication

[11] Division

[12] Prime

[13] Composite

[14] Divisibility

[15] Powers

lead to the search for *roots*.[16]

Addition, multiplication and raising to the power are indispensable for the mastery of large numbers and understanding systems of numeration. Goutard notes that ‘1963’ is nothing but the sum of products of powers of ten:

$1 \times 10^3 + 9 \times 10^2 + 6 \times 10^1 + 3 \times 10^0$

She shows how, when this is understood, even a child of six can work in scales other than that of ten. In this way she argues, “mathematical structures are revealed from one insight to the next, forming a continual progression.” (Goutard, 1963, p. 12)

[16] Roots

Table 1: *Medium Term Plan based on Kitchener School Board Year 1 Schedule (Sweeney, 1964)*

**Unit**	**Chapter.Ex**	**Exercises from Gattegno Mathematics (Gattegno, 1963a)**
1	1	Free play for the first week, and a few weeks at the end of each lesson for the next few weeks. Guided free play should follow. Thereafter lessons should set time aside for free writing (Gattegno, 2010b).
2		The learners should be taught the printing of letters used to name the rods *w,r,g,p,y ...* and numerals in September (during the writing period - not maths) so they will be able to write them correctly and with ease when it is time to write patterns (Goutard, 2017).
3	2.1-4	**Qualitative Work**. Review patterns from *red* to *orange* calling out the action such as *yellow* **plus** *pink* **is equivalent in length to** to *blue*. Write paper train names e.g. *yp*. Equivalence means that in certain circumstances – in this case where we are concerned with the length of a rod or two rods – we can exchange the **train** *yp* for the *blue* rod.
4		**Literal Work**. Using on words, *plus* and *equivalent length* and symbol for equivalence. Mathematicians use the $\sim$ sign in mathematical writing and read this **is equivalent to** when it is obvious that we are comparing lengths.
5	2.5-6	Review *shorter* and *longer* and introduces *bigger* and *smaller*. Write diagram names $w < r$, $y > p$. Ask how to write relationship between two dark greens – the suggestion to use = or the **equal** sign in writing often comes spontaneously.
6	2.8-9	*Seeing differences*.
7	2.10-13	*Staircase and complementaries*.
8	2.14-17	*Drill on equivalent lengths*.
9	2.18-19	*Seeing differences*.
10	2.20-21	*Seeing factors*.
11	2.22	*Readiness for division with remainders*.
12	2.22-30	*Trains*. Readiness for division, multiplication and factors.
13	2.31-32	Readiness for associative and commutative principles.
14	2.33-36	Background for unknown in equations
15	2.37-39	Odd and Even lengths
16	3.1-2	Teach subtraction as difference between two rods using the word **minus** using letter names for the rods.
17	3.3-7	*Staircases and equivalent differences* Various step sizes.
18	3.8-9	*Equivalent expressions*.
19	3.10-12	*Equations* with an unknown term.
20	3.13-14	*Brackets*.
21	3.15	*Writing multiples of two*.
22	3.26-27	*Multiples 3-5*.
23	3.18-19	*Multiples 6-10*.
24	3.20	*Names of Fractions*.
25	3.21	Understanding Relationships and *Writing Equivalences*.
26	4.1	**Quantitative Work** *Numerical names for the rods, measured with a white*.
27		Patterns - making *written patterns* (addition and subtraction). These exercises are not designed to learn addition and subtraction facts, but how to write numerical patterns for complementary pairs
28	4.2	Here mastery is required. *Complete Patterns for the red rod*.
29	4.3-4	*Introducing minus sign for numbers and subtraction*. Complete pattern for *light green*.
30	4.5-7	(ignore brackets in question 6). Complete pattern for *pink*. Including addition, subtraction and equations.

Table 2: *Medium Term Plan ctd.*

Unit	Chapter.Ex	Exercises from Gattegno Mathematics
31	4.8-10	Complete pattern for *yellow*. Including addition, subtraction and equations. Introducing brackets.
32a	4.11-16	Introducing multiplication and $\times$ sign.
33	1.1-4	*Multiples of 10* (Gattegno, 1963b).
34	1.8-11	*Numbers to 99* Numbers $10 < 99$ as sum of a multiple of orange and a rod. **Place Value** as encoding number of times an orange rod appears in the train (Gattegno, 1963b).
35	3.1-4	Clock and counting minutes in units of 5 *Multiples of 5* (Gattegno, 1963b).
36	4.17-18	Working with equations including $\times$ sign.
37	4.19-26	Introduce *half*. Learn how to read equations involving $\frac{1}{2}$ and $\frac{3}{2}$.
38	4.27-29	Introduce $\frac{1}{3}$
39	4.30-31	Introduce $\frac{1}{4}$
40	4.32-34	Drill and Review
41	4.35	Introduce $\frac{1}{5}$
42	4.36-38	Drill on "what fraction is one rod of another?," "What is the white of the red?" etc
43	4.39,40 42,43	Equations involving fractions and division
44	4.44	*Equivalent expressions*.
45	4.45-6	Complete pattern for *dark green*.
46	4.47-9	Introduce $\frac{1}{6}$. In exercise 54 you can compare each term with the dark green e.g. $\frac{2}{6} + \frac{3}{6} = \frac{5}{6}$
47	4.50-53	Introducing crossed rods to show multiplication and also factors
48	4.41,53	Introducing *division*. Do it orally at first and when understood introduce written work for division e. $\begin{array}{r} 3 \\ 2\overline{)6} \end{array}$
49	4.54-55	Complete pattern for the *black* rod
50	4.56-7	Pattern for *brown* (Gattegno *tan*) rod.
51	4.58-9	Pattern for *blue* rod.
52	4.60-62	Pattern for *blue* rod.
53	4.63-4	Review of 7 to 10.
54	4.65	Oral drill on fractions.
55	4.66-73	More exercises with staircases.
56	4.74	When other rods are called 1. Use Chapter 5 Applications for free writing.
57	6.1-5	Study of numbers up to 20
58	6.6,7	Study of 11
59	6.8,9	Study of 12
60	6.10	Factors of 12
61	6.11,12	Study of 13
62	6.14	Study of 14
63	6.15-17	Factors of 14
64	6.18	Study of 15
65	6.19-21	Factors and division of 15
66	6.22	Exercises with 15
67	6.23	Study of 16
68	6.24-5	Factors and division of 16
69	6.26	Exercises with 16
70	6.27-8	Study of 17
71	6.29-31	Study of 18
72	6.32-33	Study of 19

Table 3: *Medium Term Plan concluded.*

Unit	Chapter.Ex	Exercises from Gattegno Mathematics
73	6.34-37	Study of 20
74	6.38	Odds and Evens
75	6.39-40	Factors and *Prime Numbers*
76	6.41-2	*Order, Ordinality*
77	6.43-4	Arithmetic Progressions
78	6.45	Introducing Zero
79	6.46-7	Products less than 20
80	6.48-52	Short Division

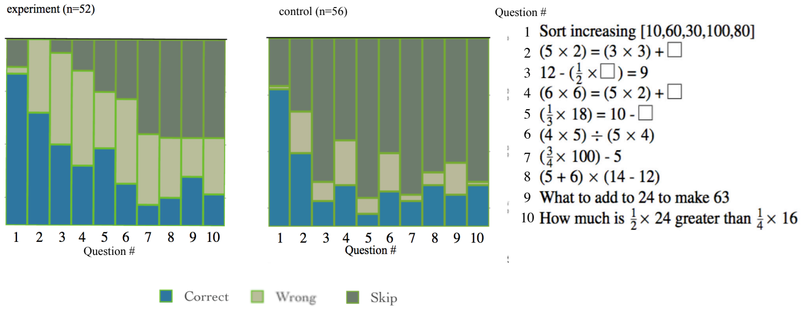

Figure 3: Percentage cumulative accuracy and attempt data for CUI test by Question ($g4$)

2. CUI data

The percentage cumulative accuracy, skip and failed attempts for the 10 question CUI test are shown in the Figure 3.

References

ATM (1977). *Notes on Mathematics for Children*. Association of Teachers of Mathematics.

Cane, J. (2017). Mathematical journeys: Our journey in colour with Cuisenaire rods. *Mathematics Teaching*, *257*, 7–11.

Choquet, G. (1963). What is modern mathematics? https://issuu.com/eswi/docs/1162_what-is-modern-mathematics.

Gattegno, C. (1960, 2010b). *Now Johnny Can Do Arithmetic: A Handbook on the use of Coloured Rods*. Educational Explorers, Fishguard.

Gattegno, C. (1963a). *Mathematics with Numbers in Colour: Numbers from 1 to 20*, volume I. Educational Explorers, Fishguard.

Gattegno, C. (1963b). *Mathematics with Numbers in Colour: Numbers to 1000 and the four operations*, volume II. Educational Explorers.

Gattegno, C. (1974, 2010a). *Common Sense of Teaching Mathematics*. Educational Solutions, New York.

Goutard, M. (1963). *Talks For Primary School Teachers*. Educational Explorers.

Goutard, M. (1964, 2017). *Mathematics and Children*. Educational Explorers, Fishguard.

Sweeney, J. R. (1964). An experimental study comparing the Cuisenaire method with traditional methods in grade 1 mathematics. In *Canadian experience with Cuisenaire method.* (pp. 117–137). Canadian Council for Research in Education.
